# Association of Diet and Physical Activity With All-Cause Mortality Among Adults With Parkinson Disease

**DOI:** 10.1001/jamanetworkopen.2022.27738

**Published:** 2022-08-19

**Authors:** Xinyuan Zhang, Samantha A. Molsberry, Michael A. Schwarzschild, Alberto Ascherio, Xiang Gao

**Affiliations:** 1 Department of Nutritional Sciences, The Pennsylvania State University, University Park; 2 Channing Division of Network Medicine, Brigham and Women’s Hospital and Harvard Medical School, Boston, Massachusetts; 3 Department of Nutrition, Harvard T.H. Chan School of Public Health, Boston, Massachusetts; 4 Social & Scientific Systems Inc, Durham, North Carolina; 5 Department of Neurology, Massachusetts General Hospital, Boston; 6 Department of Epidemiology, Harvard T.H. Chan School of Public Health, Boston, Massachusetts; 7 Department of Nutrition and Food Hygiene, School of Public Health, Institute of Nutrition, Fudan University, Shanghai, China; 8 Key Laboratory of Public Health Safety of Ministry of Education, Fudan University, Shanghai, China

## Abstract

**Importance:**

Greater diet quality and physical activity level are associated with a lower risk of developing Parkinson disease (PD). However, information regarding the association between lifestyle behaviors and survival after PD diagnosis remains limited.

**Objective:**

To examine the association of prediagnosis and postdiagnosis overall diet quality and physical activity with all-cause mortality among individuals with PD.

**Design, Setting, and Participants:**

This population-based cohort study analyzed male participants in the Health Professionals Follow-up Study from 1986 to 2012 and female participants in the Nurses’ Health Study from 1984 to 2012. Participants who were diagnosed with PD and had complete baseline dietary assessment data were included. Data were analyzed from January 2021 to February 2022.

**Exposures:**

Prediagnosis diet quality, assessed by the Alternative Healthy Eating Index (AHEI), and physical activity, assessed by metabolic equivalent task (MET) hours per week reported on questionnaires, were the primary exposures of interest to minimize reverse causation.

**Main Outcomes and Measures:**

Mortality, which was followed up until 2018, was the primary outcome. Cox proportional hazards regression models were used to estimate the association of diet and physical activity with mortality individually and jointly, and the models were adjusted for age, total energy intake, caffeine intake, and other lifestyle risk factors.

**Results:**

The sample comprised 1251 individuals with PD, which included 652 men (52.1%) with a median (IQR) age at diagnosis of 73.4 (67.5-78.7) years. During the 32 to 34 years of follow-up, 942 participants died. The adjusted hazard ratio (HR) comparing the highest vs the lowest AHEI quartile was 0.69 (95% CI, 0.56- 0.85) for prediagnosis analyses and 0.57 (95% CI, 0.42-0.78) for postdiagnosis analyses. Similar results were obtained for cumulative mean MET hours per week in the prediagnosis analyses (HR, 0.71; 95% CI, 0.57-0.87) and postdiagnosis analyses (HR, 0.47; 95% CI, 0.35-0.63). The inverse association persisted for PD-specific mortality (postdiagnosis AHEI: HR, 0.52 [95% CI, 0.33-0.80]; postdiagnosis physical activity: HR, 0.37 [95% CI, 0.25-0.55]). In the joint analyses of diet quality and physical activity before the PD diagnosis, the adjusted HR was 0.51 (95% CI, 0.36-0.73) for individuals in the highest vs lowest tertiles for both variables. The HR for diet quality and physical activity after the diagnosis was 0.35 (95% CI, 0.23-0.52).

**Conclusions and Relevance:**

Results of this study showed that a healthy dietary pattern and an active lifestyle were associated with a lower rate of all-cause mortality among individuals with PD. Consuming a healthy diet and engaging in physical activity or exercise could be targeted to improve PD outcomes.

## Key Points

**Question:**

What is the association of diet and physical activity with survival among individuals with Parkinson disease (PD)?

**Findings:**

In this cohort study of 1251 confirmed cases of PD, a better diet quality, such as following the Alternative Healthy Eating Index, and a higher level of physical activity both before and after PD diagnosis were inversely and independently associated with mortality risk. Individuals in the highest groups of both diet and physical activity had the lowest mortality rate, although no interaction was found between diet and physical activity.

**Meaning:**

Findings from this study suggest that consuming a healthy diet and engaging in physical activity could improve PD outcome.

## Teaser

This cohort study examines the dietary patterns, activity levels, and survival outcomes in individuals with Parkinson disease before and after their diagnosis.

## Introduction

Lifestyle behaviors, including diet and physical activity, have been associated with the risk of developing Parkinson disease (PD), the second most common neurodegenerative disease.^1^ For example, better diet quality, as assessed by the Alternate Healthy Eating Index (AHEI) and the alternate Mediterranean Diet (aMED) Score, and higher levels of physical activity were associated with a lower risk of incident PD^2,3,4,5^ and prodromal PD symptoms.^6,7^ However, little is known regarding their long-term role in PD survival.^8^ Few prospective studies have assessed the combined outcome of diet and physical activity associated with all-cause mortality,^9^ and none of these studies specifically focused on individuals with PD.

Despite improvements in the clinical management of the motor symptoms of PD, there is little evidence that current treatments slow the progressive neuronal loss. Consensus guidelines on complementary, disease-modifying lifestyle behaviors are lacking, which could partly be attributed to insufficient evidence from prospective studies and randomized clinical trials.^10^ Thus, we aimed to examine the association of prediagnosis and postdiagnosis overall diet quality and physical activity with all-cause mortality among individuals with PD from 2 large US cohorts, the Nurses’ Health Study (NHS) and the Health Professionals Follow-up Study (HPFS). We hypothesized that better diet quality and higher level of physical activity individually and jointly contribute to better PD survival.

## Methods

The study protocol for this population-based prospective cohort study was approved by the institutional review boards of Brigham and Women’s Hospital, Harvard T.H. Chan School of Public Health, and the participating state cancer registries as required. These institutional review boards allowed the completion of questionnaires to serve as implied consent from participants. We followed the Strengthening the Reporting of Observational Studies in Epidemiology (STROBE) reporting guideline.

### Study Population

The NHS was established in 1976 with 121 700 female registered nurses aged 30 to 55 years, and the HPFS was established 10 years later with 51 529 male veterinarians, dentists, pharmacists, optometrists, osteopath physicians, and podiatrists aged 40 to 75 years. Participants in both cohorts reported information on lifestyle behaviors, disease, and medical history via mailed questionnaires at baseline and every 2 years afterward.

Participants with confirmed PD were eligible for the present study. Because the most comprehensive diet assessment started in 1984 in the NHS, we used the 1984 questionnaire return as the baseline for the NHS and 1986 as the baseline for HPFS. Cases of PD in the NHS and HPFS were identified from the biennial questionnaires until 2012 and confirmed via medical records review by a movement disorder specialist, as described in a previous study.^11^ Briefly, potential cases of PD were first identified from the self-reported questionnaires, and the treating neurologists were contacted to either confirm the diagnosis or send a copy of the patients’ medical records. The movement disorder specialist then evaluated the clinical certainty of a PD diagnosis.

Participants who had a history of PD or had missing diet assessment data at the study baseline were excluded. The final sample for the current analysis included 652 men from the HPFS and 599 women from the NHS.

### Assessment of Diet Quality

Diet information was obtained from responses to validated semiquantitative food frequency questionnaires in 1984 (NHS), 1986 (NHS and HPFS), and every 4 years thereafter in both cohorts.^12,13^ Overall diet quality was assessed using the AHEI, which was developed and validated in the NHS and HPFS cohorts and was based on foods and nutrients associated with chronic disease risk^14^ as well as major chronic diseases (including PD,^2,6^ cardiovascular diseases, and cancer) and mortality.^15^ The AHEI consists of 11 components, and each component is scored from 0 (indicating worst diet quality) to 10 (indicating best diet quality).^14^ Better scores are given to a higher intake of vegetables, fruits, whole grains, nuts and legumes, long-chain omega-3 fatty acids, and polyunsaturated fatty acids; a lower intake of sugar-sweetened beverages and fruit juices, red or processed meats, trans fat, and sodium; and a light to moderate intake of alcohol. Thus, the total AHEI ranges from 0 to 110.

The aMED Score, another common diet quality measure, was used as a secondary exposure in the current analysis. The aMED Score consists of 9 components, and each component is scored as 0 (indicating nonadherence) or 1 (indicating adherence).^16^ Adherence corresponds to a higher intake of vegetables, fruits, legumes, nuts, whole grains, and fish and the ratio of monounsaturated fat to saturated fat; a lower intake of red or processed meats; and a light to moderate intake of alcohol. The total aMED Score thus ranged from 0 to 9.

### Assessment of Physical Activity

Physical activity was assessed from responses to validated physical activity questionnaires every 2 to 4 years in both NHS and HPFS cohorts.^17,18^ Briefly, the questionnaires asked about the usual time spent weekly on 8 to 10 activities, including walking, jogging, running, swimming, bicycling, calisthenics, racquet sports, tennis (in NHS), weightlifting, outdoor work (in HPFS), and climbing stairs, during the past year. The weekly expenditure of metabolic equivalent of sitting quietly for an hour (metabolic equivalent task [MET] h/wk) was calculated for each of these activities and total activity.^19^ The questionnaire measurements showed good correlation with diary-assessed activity scores (which ranged from 0.28-0.58, with the lowest score indicating inactivity and the highest score indicating vigorous activity).^17,18^

### Ascertainment of Mortality

The primary outcome of this study was mortality. Mortality information until 2018 was collected from the National Death Index, the US Postal Service, or family members and was confirmed by reviewing death certificates or related medical records. More than 97% of deaths were identified, and records matched by the National Death Index had a sensitivity of 97% and specificity of 100%.^20,21^ Parkinson disease–specific mortality, the secondary outcome of this study, was identified using *International Classification of Diseases, Eighth Revision* diagnosis codes for the NHS cohort and *International Classification of Diseases, Ninth Revision* diagnosis codes for the HPFS cohort.

### Assessment of Covariates

Data on smoking status, body mass index (BMI), nonsteroidal anti-inflammatory drug use, chronic disease history, and postmenopausal hormone use in women were collected at baseline and every 2 years from the self-reported biennial questionnaires. Smoking status was categorized according to cumulative pack-years. Body mass index was calculated as weight in kilograms divided by height in meters squared. Total energy intake and the energy-adjusted caffeine intake were ascertained at baseline and every 4 years using the food frequency questionnaire.^22,23^

### Statistical Analysis

Because of the potential decrease in life quality associated with the onset of classic motor symptoms of PD (eg, tremor, dysphagia, and other motor abnormalities) that affect dietary and physical behaviors,^24^ we examined prediagnosis diet quality and physical activity as the primary exposures of interest to minimize reverse causation. We also examined postdiagnosis diet quality and physical activity for a typical time-to-event analysis, with the date of diagnosis as the start of follow-up.

In the prediagnosis analyses, diet quality and physical activity were calculated as the cumulative mean of all assessments from baseline to the current cycle until the last questionnaire return and before the PD diagnosis date. Values were no longer updated after the diagnosis. Time and person-years were calculated from baseline to death or the end of follow-up. All covariates were correspondingly updated. Using time-dependent variables and calculating the cumulative means allowed us to characterize the long-term exposure status and to lower the immortal time bias in time-to-event analyses.^25^

In the postdiagnosis analyses, variables were calculated as the cumulative mean of assessments from the questionnaire with the earliest return date after the PD diagnosis date to the current cycle until death or the end of the study. Prediagnosis exposure levels were further adjusted in postdiagnosis analyses specifically to evaluate the associations independent of prediagnosis diet and physical activity levels. Time and person-years were calculated from the return of the first questionnaire after diagnosis date to death or end of follow-up.

Cox proportional hazards regression models were used to estimate hazard ratios (HRs) with 95% CIs for all-cause mortality. We tested the proportional hazard assumption by including in the models the multiplication term of exposures and the time scale. We categorized the cumulative mean of AHEI, aMED Score, and MET hours per week into cohort-specific quartiles and used the lowest quartile as the reference. Physical activity and diet quality scores were mutually adjusted. The median values of each quartile were used to test for linear trends. Age, smoking status (never smoked, <10 pack-years, 10-24 pack-years, 25-44 pack-years, or ≥45 pack-years), BMI (<21, 21-24.9, 25-29.9, or ≥30), total energy intake (kilocalorie [kcal]/d, in quartiles), caffeine intake (mg/d, in quartiles), nonsteroidal anti-inflammatory drug use (yes or no), hypertension (yes or no), type 2 diabetes (yes or no), hypercholesterolemia (yes or no), and postmenopausal hormone use in women (premenopausal or never used, current user, or past user) were adjusted in the model. In addition, we categorized the exposures into tertiles, cross-classified the participants into 9 categories (AHEI tertiles 1, 2, and 3 × MET hours per week tertiles 1, 2, and 3), and tested the joint effect and additive interaction of diet and physical activity.^26,27^ Race and ethnicity data were not analyzed because participants in these cohorts were predominantly White individuals.

Analyses were conducted separately in each cohort, and HRs were pooled using random-effects models. To estimate the role of unmeasured confounders, we calculated E-values for the main models.^28^ To further account for the potential of reverse causation, we conducted 4-year lagged analyses, wherein we stopped updating the cumulative mean of exposures 4 years before PD diagnosis in prediagnosis analyses and 4 years before death or end of follow-up in postdiagnosis analyses. We also tested whether the results were modified by smoking status or BMI by including a multiplication term in the model, after adjusting for covariates.

All analyses were conducted in SAS, version 9.2 for UNIX (SAS Institute Inc), from January 2021 to February 2022. Two-sided *P* < .05 was considered to be statistically significant.

## Results

A total of 1251 confirmed cases of PD were included in this study. Of this sample, 652 (52.1%) were men from the HPFS with a median (IQR) age at diagnosis of 73.4 (67.5-78.7) years and 599 (47.9%) were women from the NHS, with a median (IQR) age at diagnosis of 71.9 (66.5-77.6) years. Characteristics of participants at the last questionnaire return before PD diagnosis are presented in Table 1 according to the AHEI quartiles and in eTable 1 in the Supplement according to the MET hours per week quartiles. Participants who had a healthier diet also had higher physical activity levels, were likely to smoke less, had lower BMI, lower caffeine intake, and higher flavonoid intake.

**Table 1.** Characteristics of Participants at the Last Questionnaire Return Before Parkinson Disease Diagnosis

	Participants, No. (%)			
	AHEI quartile 1	AHEI quartile 2	AHEI quartile 3	AHEI quartile 4
**Health Professionals Follow-up Study**				
No. of participants	163	163	163	163
Age at diagnosis, mean (SD), y^a^	69.9 (8.9)	73.7 (8.2)	73 (8.2)	74.7 (7.2)
AHEI, mean (SD)	43 (5.0)	53.8 (3.0)	61.8 (2.2)	74.1 (6.0)
aMED Score, mean (SD)	2.9 (1.6)	4.1 (1.6)	4.8 (1.5)	5.9 (1.5)
Physical activity, mean (SD), MET h/wk^b^	22.9 (26.1)	23.5 (24.9)	29.5 (32.1)	39.6 (33.4)
Smoking status				
Never	93 (53.6)	93 (54.9)	88 (54.5)	102 (61.7)
Past	67 (45.1)	66 (42.0)	70 (42.9)	61 (38.3)
Current	3 (1.4)	4 (3.0)	5 (2.6)	0
BMI, mean (SD)	26.3 (3.1)	25.8 (2.9)	25.4 (2.8)	24.4 (2.9)
Total energy intake, mean (SD), kcal/d	2046 (668.5)	2019 (699.9)	1905 (632.2)	1850 (567.6)
Caffeine intake, mean (SD), mg/d	182.7 (187.6)	146.5 (206.8)	135 (161.4)	100.3 (112.8)
Total flavonoids intake, mean (SD), mg/d	269.5 (220.9)	307.1 (256.7)	344.5 (216.0)	428.7 (232.0)
Use of NSAIDs	38 (24.3)	44 (27.0)	50 (30.1)	58 (36.1)
Hypertension	66 (41.7)	65 (39.6)	77 (46.5)	82 (48.7)
Diabetes	13 (9.0)	14 (8.2)	14 (9.0)	14 (6.2)
Hypercholesterolemia	55 (34.2)	78 (47.8)	67 (43.4)	88 (52.6)
**Nurses’ Health Study**				
No. of participants	149	150	150	150
Age at diagnosis, mean (SD), y^a^	71.3 (7.4)	70.8 (7.7)	72.8 (7.6)	72.6 (7.7)
AHEI, mean (SD)	41.1 (4.4)	50.5 (2.2)	58.3 (2.6)	70.2 (5.1)
aMED Score, mean (SD)	2.8 (1.5)	3.9 (1.6)	4.7 (1.7)	5.5 (1.5)
Physical activity, mean (SD), MET h/wk^b^	9.9 (12.9)	14.8 (17.8)	17.1 (15.8)	21.4 (20.2)
Smoking status				
Never	87 (59.5)	87 (58.5)	90 (60.9)	66 (42.2)
Past	53 (35.4)	56 (38.0)	58 (37.5)	81 (55.2)
Current	9 (5.1)	7 (3.6)	2 (1.6)	3 (2.5)
BMI, mean (SD)	25.9 (4.4)	26 (4.2)	25.8 (4.7)	25.5 (4.6)
Total energy intake, mean (SD), kcal/d	1698 (527.0)	1716 (541.0)	1774 (584.1)	1626 (520.9)
Caffeine consumption, mean (SD), mg/d	138.9 (181.3)	139.9 (163.2)	176.7 (163.8)	155.8 (177.9)
Total flavonoids intake, mg/d	285 (232.9)	340.2 (366.1)	380.3 (327.8)	484 (453.5)
Use of NSAIDs	38 (26.5)	46 (31.6)	35 (22.7)	51 (34.3)
Hypertension	69 (45.8)	89 (62.6)	77 (52.0)	71 (51.7)
Diabetes	10 (7.6)	11 (8.2)	10 (5.6)	11 (8.8)
Hypercholesterolemia	84 (56.0)	86 (61.5)	97 (63.8)	102 (70.2)
Postmenopausal hormone use				
Premenopausal; never	54 (37.2)	62 (42.2)	49 (30.3)	39 (29.2)
Current	39 (25.1)	40 (25.0)	38 (26.7)	36 (23.3)
Past	56 (37.8)	48 (32.7)	63 (43.0)	75 (47.5)

Abbreviations: AHEI, Alternative Healthy Eating Index; aMED, alternate Mediterranean Diet; BMI, body mass index (calculated as weight in kilograms divided by height in meters squared); kcal, kilocalorie; MET, metabolic equivalent task; NSAID, nonsteroidal anti-inflammatory drug.

^a^ Value was not age standardized.

^b^ MET hours per week from recreational and leisure-time activities.

During the 32 to 34 years of follow-up, 942 participants died (529 men and 413 women). Both prediagnosis and postdiagnosis diet qualities were inversely associated with mortality risk in individuals with PD. In the pooled analysis, compared with the lowest quartile of prediagnosis cumulative mean AHEI score, the multivariable-adjusted HR of the highest quartile was 0.69 (95% CI, 0.56- 0.85; *P* for trend = .002) (Table 2), without a significant difference between men and women. The E-value of 1.91 suggests that an unmeasured confounder must be associated with both the exposure and the outcome with a relative risk greater than 1.91 to fully explain the current association. This association was attenuated in the 4-year lagged analyses (HR, 0.76; 95% CI, 0.61-0.95; *P* for trend = .02) (Table 2). The magnitude of an inverse association was stronger in the postdiagnosis analyses even after controlling for prediagnosis diet quality (HR, 0.57; 95% CI, 0.42-0.78; *P* for trend < .001; *P* for heterogeneity = .79) (Table 2). In the postdiagnosis but not the prediagnosis analyses, higher adherence to the aMED was also inversely associated with mortality rate in PD (HR, 0.65; 95% CI, 0.48-0.87; *P* for trend = .009) (eTable 2 in the Supplement).

**Table 2.** Association Between Diet Quality and Mortality Risk Among Individuals With Parkinson Disease, According to the AHEI

	HR (95% CI)				*P* value for trend
	AHEI quartile 1 (worst diet quality)	AHEI quartile 2	AHEI quartile 3	AHEI quartile 4 (best diet quality)	
**Prediagnosis analyses, cumulative mean**					
Health Professionals Follow-up Study					
AHEI, median (IQR)	42.5 (38.6-45.4)	51.7 (49.7-53.6)	58.8 (57.0-60.2)	67.1 (64.0-70.4)	NA
PD cases, No.	120	130	133	146	NA
Incidence per 100 000 person-years	3049	3291	3361	3715	NA
Age-adjusted model	1 [Reference]	0.75 (0.57-0.98)	0.66 (0.51-0.86)	0.72 (0.55-0.94)	.01
Multivariable-adjusted model^a^	1 [Reference]	0.78 (0.59-1.04)	0.68 (0.52-0.90)	0.74 (0.56-0.98)	.05
4-y Lagged	1 [Reference]	0.81 (0.60-1.08)	0.68 (0.51-0.91)	0.78 (0.58-1.05)	.11
Nurses' Health Study					
AHEI, median (IQR)	40.6 (37.0-43.4)	48.6 (46.7-50.2)	54.8 (53.1-56.7)	63.6 (60.8-67.7)	NA
PD cases, No.	93	100	112	108	NA
Incidence per 100 000 person-years	2224	2396	2683	2585	NA
Age-adjusted model	1 [Reference]	0.73 (0.54-0.98)	0.82 (0.62-1.10)	0.62 (0.46-0.83)	.005
Multivariable-adjusted model^a^	1 [Reference]	0.76 (0.56-1.02)	0.86 (0.63-1.17)	0.63 (0.46-0.86)	.01
4-y Lagged	1 [Reference]	0.91 (0.67-1.25)	0.98 (0.71-1.36)	0.74 (0.53-1.03)	.07
Pooled					
Multivariable-adjusted model^a^	1 [Reference]	0.77 (0.62-0.95)	0.76 (0.61-0.95)	0.69 (0.56-0.85)	.002
4-y Lagged	1 [Reference]	0.86 (0.69-1.06)	0.81 (0.57-1.16)	0.76 (0.61-0.95)	.02
**Postdiagnosis analyses, cumulative mean**					
Health Professionals Follow-up Study					
AHEI, median (IQR)	44.5 (39.6-47.2)	54.4 (52.1-56.7)	62.4 (60.4-64.6)	72.7 (69.5-77.8)	NA
PD cases, No.	120	113	129	101	NA
Incidence per 100 000 person-years	8685	8087	9231	7261	NA
Age-adjusted model	1 [Reference]	0.84 (0.63-1.12)	0.76 (0.57-1.01)	0.55 (0.40-0.75)	<.001
Multivariable-adjusted model^a^	1 [Reference]	0.77 (0.57-1.04)	0.73 (0.54-1.00)	0.51 (0.37-0.71)	<.001
Multivariable and prediagnosis level	1 [Reference]	0.85 (0.62-1.18)	0.84 (0.58-1.21)	0.55 (0.36-0.84)	.003
4-y Lagged	1 [Reference]	0.79 (0.55-1.13)	0.65 (0.43-0.98)	0.50 (0.31-0.80)	.002
Nurses' Health Study					
AHEI, median (IQR)	41.6 (38.0-44.6)	50.8 (48.9-52.7)	57.5 (55.7-60.0)	68.3 (64.2-73.4)	NA
PD cases, No.	103	105	93	67	NA
Incidence per 100 000 person-years	7123	7227	6370	4620	NA
Age-adjusted model	1 [Reference]	0.90 (0.68-1.20)	0.75 (0.55-1.02)	0.50 (0.36-0.70)	<.001
Multivariable-adjusted model^a^	1 [Reference]	1.00 (0.74-1.35)	0.82 (0.59-1.13)	0.59 (0.41-0.84)	.001
Multivariable and prediagnosis level	1 [Reference]	0.95 (0.69-1.32)	0.80 (0.55-1.17)	0.60 (0.37-0.95)	.02
4-y Lagged	1 [Reference]	0.91 (0.63-1.31)	0.88 (0.58-1.35)	0.56 (0.33-0.94)	.03
Pooled					
Multivariable and prediagnosis level	1 [Reference]	0.90 (0.72-1.14)	0.82 (0.63-1.07)	0.57 (0.42-0.78)	<.001
4-y Lagged	1 [Reference]	0.84 (0.65-1.09)	0.76 (0.56-1.02)	0.52 (0.37-0.74)	<.001

Abbreviations: AHEI, Alternative Healthy Eating Index; HR, hazard ratio; NA, not applicable; PD, Parkinson disease.

^a^ Multivariable model was adjusted for age (year), physical activity (metabolic equivalent task h/wk, in quartiles), smoking status (never, <10 pack-years, 10-24 pack-years, 25-44 pack-years, or ≥45 pack-years), body mass index (calculated as weight in kilograms divided by height in meters squared; <21, 21-24.9, 25-29.9, or ≥30), total energy intake (kcal/d, in quartiles), caffeine intake (mg/d, in quartiles), nonsteroidal anti-inflammatory drug use (yes or no), hypertension (yes or no), type 2 diabetes (yes or no), hypercholesterolemia (yes or no), and postmenopausal hormone use in women (premenopausal/never, current, or past).

As expected, the observed association of diet quality with mortality was attenuated but remained significant when further adjusting for total flavonoid intake in the prediagnosis analyses (HR, 0.72; 95% CI, 0.52-1.01; *P* for trend = .07) and postdiagnosis analyses (HR, 0.60; 95% CI, 0.44-0.82; *P* for trend < .001) (eTable 3 in the Supplement). Among the individual components of AHEI, we found that following the recommendation of higher intake of whole grains (HR, 0.56; 95% CI, 0.45-0.71) and nuts and legumes (HR, 0.78; 95% CI, 0.62-0.98) as well as lower intake of red or processed meats (HR, 0.69; 95% CI, 0.52-0.92) was associated with lower mortality rate in the prediagnosis analyses (eTable 4 in the Supplement).

Table 3 showed a similar inverse association between physical activity and mortality in PD. In the pooled analysis compared with the lowest quartile of prediagnosis cumulative mean MET hours per week the multivariable-adjusted HR in the highest quartile was 0.71 (95% CI, 0.57-0.87; *P* for trend = .004; *P* for heterogeneity = .90) (Table 3). The pooled E-value was 1.85. This inverse association remained in the 4-year lagged analyses (HR, 0.68; 95% CI, 0.52-0.88; *P* for trend < .001) (Table 3). In the postdiagnosis analyses, the magnitude of the association was stronger (HR, 0.47; 95% CI, 0.35-0.63; *P* for trend < .001; *P* for heterogeneity = .44) (Table 3).

**Table 3.** Association Between Physical Activity and Mortality Risk Among Individuals With Parkinson Disease, According to MET Hours per Week

	HR (95% CI)				*P* value for trend
	MET h/wk quartile 1 (worst diet quality)	MET h/wk quartile 2	MET h/wk quartile 3	MET h/wk quartile 4 (best diet quality)	
**Prediagnosis analyses, cumulative mean**					
Health Professionals Follow-up Study					
MET h/wk, median (IQR)	4.9 (3.1-7.5)	15.1 (12.1-18.2)	28.8 (24.9-32.8)	52.2 (43.3-66.5)	NA
PD cases, No.	130	130	149	120	NA
Incidence per 100 000 person-years	3333	3292	3778	3015	NA
Age-adjusted model	1 [Reference]	0.90 (0.69-1.18)	0.94 (0.72-1.22)	0.65 (0.50-0.85)	.001
Multivariable-adjusted model^a^	1 [Reference]	0.96 (0.73-1.26)	1.03 (0.78-1.36)	0.68 (0.51-0.91)	.008
4-y Lagged	1 [Reference]	0.82 (0.61-1.09)	0.90 (0.67-1.21)	0.61 (0.45-0.83)	.003
Nurses' Health Study					
MET h/wk, median (IQR)	2.8 (1.3-3.9)	7.8 (6.5-9.5)	15.4 (13.3-17.5)	29.5 (24.2-39.7)	NA
PD cases, No.	103	102	106	102	NA
Incidence per 100 000 person-years	2470	2436	2544	2438	NA
Age-adjusted model	1 [Reference]	0.68 (0.51-0.90)	0.55 (0.41-0.73)	0.69 (0.52-0.92)	.06
Multivariable-adjusted model^a^	1 [Reference]	0.66 (0.49-0.89)	0.57 (0.42-0.77)	0.74 (0.54-1.00)	.23
4-y lagged	1 [Reference]	0.72 (0.53-0.98)	0.54 (0.40-0.74)	0.81 (0.60-1.10)	.48
Pooled					
Multivariable-adjusted model^a^	1 [Reference]	0.80 (0.55-1.15)	0.77 (0.43-1.38)	0.71 (0.57-0.87)	.004
4-y lagged	1 [Reference]	0.78 (0.63-0.97)	0.67 (0.39-1.15)	0.68 (0.52-0.88)	<.001
**Postdiagnosis analyses, cumulative mean**					
Health Professionals Follow-up Study					
MET h/wk, median (IQR)	2.5 (0.8-4.5)	13.7 (10.3-16.8)	26.9 (23.4-30.6)	51.1 (41.6-68.4)	NA
PD cases, No.	152	139	104	68	NA
Incidence per 100 000 person-years	10 895	10 102	7420	4875	NA
Age-adjusted model	1 [Reference]	1.18 (0.90-1.54)	0.87 (0.66-1.16)	0.52 (0.38-0.72)	<.001
Multivariable-adjusted model^a^	1 [Reference]	1.20 (0.90-1.59)	0.86 (0.63-1.16)	0.51 (0.36-0.71)	<.001
Multivariable and prediagnosis level	1 [Reference]	1.16 (0.86-1.55)	0.77 (0.55-1.06)	0.44 (0.30-0.64)	<.001
4-y Lagged	1 [Reference]	1.21 (0.85-1.71)	1.09 (0.75-1.60)	0.78 (0.51-1.19)	.17
Nurses' Health Study					
MET h/wk, median (IQR)	1.5 (0.8-2.3)	5.4 (4.2-6.8)	12.1 (9.8-14.6)	27.7 (21.7-37.1)	NA
PD cases, No.	121	106	80	61	NA
Incidence per 100 000 person-years	8389	7274	5500	4193	NA
Age-adjusted model	1 [Reference]	0.82 (0.62-1.08)	0.69 (0.51-0.93)	0.54 (0.39-0.75)	<.001
Multivariable-adjusted model^a^	1 [Reference]	0.83 (0.62-1.10)	0.66 (0.48-0.91)	0.54 (0.38-0.77)	<.001
Multivariable and prediagnosis level	1 [Reference]	0.81 (0.59-1.10)	0.67 (0.47-0.95)	0.52 (0.34-0.80)	.003
4-y Lagged	1 [Reference]	0.85 (0.60-1.21)	0.78 (0.52-1.16)	0.67 (0.42-1.07)	.10
Pooled					
Multivariable and prediagnosis level	1 [Reference]	0.97 (0.68-1.38)	0.72 (0.57-0.92)	0.47 (0.35-0.63)	<.001
4-y Lagged	1 [Reference]	0.95 (0.68-1.34)	0.87 (0.64-1.18)	0.69 (0.50-0.95)	.09

Abbreviations: HR, hazard ratio; MET, metabolic equivalent task; NA, not applicable; PD, Parkinson disease.

^a^ Multivariable model was adjusted for age (year), physical activity (MET h/wk, in quartiles), smoking status (never, <10 pack-years, 10-24 pack-years, 25-44 pack-years, or ≥45 pack-years), body mass index (calculated as weight in kilograms divided by height in meters squared; <21, 21-24.9, 25-29.9, or ≥30), total energy intake (kcal/d, in quartiles), caffeine intake (mg/d, in quartiles), nonsteroidal anti-inflammatory drug use (yes or no), hypertension (yes or no), type 2 diabetes (yes or no), hypercholesterolemia (yes or no), and postmenopausal hormone use in women (premenopausal/never, current, or past).

For the joint analyses, we used the same set of covariates. In the prediagnosis analyses, after pooling the results from both NHS and HPFS cohorts, greater adherence to a healthy diet and an active lifestyle (both in the highest tertile) had a 49% lower risk of mortality (HR, 0.51; 95% CI, 0.36-0.73; *P* for interaction = .19) (Table 4) compared with poor adherence and lower activity level (both in the lowest tertile). Similar results were observed in the postdiagnosis analyses (HR, 0.35; 95% CI, 0.23-0.52; *P* for interaction = .64). We did not find any significant additive interaction between diet quality and physical activity (Table 4).

**Table 4.** Joint Analysis of Diet Quality and Physical Activity for Mortality Risk Among Individuals With Parkinson Disease

	HR (95% CI)				*P* value for interaction
	MET h/wk tertile 1		MET h/wk tertile 3		
	AHEI tertile 1 (worst diet quality)	AHEI tertile 3	AHEI tertile 1	AHEI tertile 3 (best diet quality)	
Prediagnosis analyses, cumulative mean					
Health Professionals Follow-up Study					
PD cases, No. at baseline	168	78	23	53	NA
Multivariable-adjusted model^a^	1 [Reference]	0.70 (0.45-1.08)	0.72 (0.46-1.15)	0.61 (0.43-0.87)	.43
Nurses' Health Study					
PD cases, No. at baseline	149	45	44	52	NA
Multivariable-adjusted model^a^	1 [Reference]	0.61 (0.38-0.97)	0.44 (0.26-0.74)	0.42 (0.29-0.62)	.17
Pooled	1 [Reference]	0.65 (0.47-0.90)	0.57 (0.35-0.93)	0.51 (0.36-0.73)	.19
Postdiagnosis analyses, cumulative mean^b^					
Health Professionals Follow-up Study	1 [Reference]	0.52 (0.32-0.86)	0.51 (0.30-0.86)	0.36 (0.21-0.62)	.81
Nurses' Health Study	1 [Reference]	0.61 (0.36-1.02)	0.66 (0.38-1.13)	0.34 (0.19-0.60)	.54
Pooled	1 [Reference]	0.56 (0.39-0.80)	0.58 (0.39-0.84)	0.35 (0.23-0.52)	.64

Abbreviations: AHEI, Alternative Healthy Eating Index; HR, hazard ratio; MET, metabolic equivalent task; NA, not applicable; PD, Parkinson disease.

^a^ Multivariable model was adjusted for age (year), physical activity (MET h/wk, in quartiles), smoking status (never, <10 pack-years, 10-24 pack-years, 25-44 pack-years, or ≥45 pack-years), body mass index (calculated as weight in kilograms divided by height in meters squared; <21, 21-24.9, 25-29.9, or ≥30), total energy intake (kcal/d, in quartiles), caffeine intake (mg/d, in quartiles), nonsteroidal anti-inflammatory drug use (yes or no), hypertension (yes or no), type 2 diabetes (yes or no), hypercholesterolemia (yes or no), and postmenopausal hormone use in women (premenopausal/never, current, or past).

^b^ Postdiagnosis models were further adjusted for prediagnosis levels.

In the sensitivity analysis for mortality attributed to PD (308 cases in the HPFS; 211 cases in the NHS), the results remained similar (eTable 5 in the Supplement). The inverse association persisted for PD-specific mortality (postdiagnosis AHEI: HR, 0.52 [95% CI, 0.33-0.80]; postdiagnosis physical activity: HR, 0.37 [95% CI, 0.25-0.55]). We did not find interactions between the exposures and age, smoking status, or BMI.

## Discussion

To our knowledge, this cohort study has been the largest to date that examined the association of overall diet quality and physical activity with survival among individuals with PD in prospective cohorts. Following a healthier dietary pattern, such as the AHEI, and participating in frequent physical activities both before and after PD diagnosis were associated with lower mortality rate. Even after controlling for prediagnosis levels, postdiagnosis diet quality and physical activity were still significantly associated with lower mortality rate.

Although the health benefits of overall diet quality and physical activity have been well documented in epidemiological studies, few of such studies have focused on individuals with PD, a population with complex motor and nonmotor symptoms who grapple with lack of treatment effectiveness, compromised quality of life, and high risk of mortality. A cohort study of 360 individuals with PD identified several lifestyle factors associated with mortality.^29^ For example, a history of competitive sports (eg, basketball) was associated with lower mortality risk (HR, 0.71; 95% CI, 0.52-0.99) and having total MET hours above the median showed a pattern of lower risk (HR, 0.82; 95% CI, 0.60-1.12) compared with total MET hours below the median.^29^ However, this study did not have a comprehensive measure of dietary intake. Another cohort study of 699 individuals with PD found a dose-response association between physical activity and all-cause mortality.^8^ The HR comparing the highest with lowest total MET minutes per week quartile was 0.61 (95% CI, 0.53-0.70), which is similar to the risk reduction found in the present study.

The results of this study suggest the need for more research into lifestyle behaviors among individuals with PD. Compared with previous studies, the present study comprehensively assessed the quality of both diet and physical activity and tested the interaction between these modifiable lifestyle factors. We found that both prediagnosis and postdiagnosis healthy diet and physical activity were associated with mortality outcome. This finding suggests that consuming a high-quality diet and engaging in physical activity are beneficial for survival not only before PD symptom onset but also after PD diagnosis, independent of prediagnosis levels.

In line with the results of most prospective studies that reported benefits of healthy dietary patterns for PD risk,^1,3^ findings of this study showed that the highest level of adherence to a healthy diet (eg, AHEI score >59-67, corresponding to the range of cutoff values for the highest quartiles) had the lowest mortality risk. A comprehensive diet quality score captures not only the intake level of each component of that score but also higher-level interactions. We hypothesized several mechanism explanations.

First, greater diet quality, as assessed by the AHEI and aMED Score, featured higher consumption of fruits and vegetables that are high in antioxidants. We found that the inverse association between diet quality and mortality was attenuated when total flavonoid intake was further adjusted in the models. This finding is supported by a previous report that a high intake of flavonoids and flavonoid-rich foods, such as berries and red wines, both before and after PD diagnosis, was associated with lower mortality risk.^30^ Second, healthier dietary patterns were associated with reduced oxidative stress and consequent metabolic risk factors for PD.^31,32^ Oxidative stress and neuroinflammation are among the crucial players in the pathophysiological process of neurodegeneration.^32^ Third, diet could modify the gut microbiome and can affect gene expression by inducing epigenetic modifications,^33^ but the relevance of these mechanisms to PD progression and mortality is unknown.^34,35^ In the current study, the protective properties of AHEI seemed to be stronger than protection from aMED Score, but we cannot exclude the possibility of a chance finding.

Compared with less active participants in each cohort, men expending at least 35 to 37 MET hours per week and women expending at least 18 to 21 MET hours per week (corresponding to the range of cutoff values for the highest quartiles) of leisure-time physical activity either before or after PD diagnosis had the lowest mortality risk. This time translates to approximately 10 to 20 hours of walking or approximately 5 to 9 hours of moderate-intensity activities per week.^19^ Mechanisms that have been proposed to explain the neuroprotective properties of physical activity include reduction of chronic inflammation,^36^ protection against mitochondrial dysfunction,^37^ and expression of growth factors and receptors.^4^ Experiments in animal models found an association between vigorous exercise and mitigated effects of dopaminergic neurotoxins as well as increased brain-derived neurotrophic factors.^38^ Because of the common motor symptoms such as tremor, rigidity, and nonmotor symptoms such as sleep disorders and depression, individuals with PD tend to lead a sedentary lifestyle,^39^ but education and promotion on proper activity level are preferred.

Results of this study suggest that a healthier diet and an active lifestyle, both before and after the PD diagnosis, provide independent, plausible protection against mortality. These results could inform the design of clinical trials and the development of guidelines and recommendations for adopting a healthy lifestyle in individuals with PD or with prodromal symptoms of PD. Contrary to our hypothesis, we did not find a statistically significant interaction between diet quality scores and total MET hours per week, suggesting that diet and physical activity may be independent factors in PD mortality. Survival is an important aspect of a good outcome in PD. Despite progress in understanding the pathophysiological process of PD, effective disease management remains elusive. A meta-analysis found that almost all 54 included studies reported higher mortality in patients with PD compared with control participants, and the pooled mortality ratio in the inception cohorts was 1.41 (95% CI, 1.28-1.55).^40^ Furthermore, the studies showed an increasing pattern of mortality ratios in PD over time, suggesting the need for greater awareness of PD survival.^40^

### Strengths and Limitations

This study has some strengths. Among these strengths are the large sample size, long follow-up duration, and repeated assessments of lifestyle factors using well-validated instruments. We also carefully adjusted for well-established risk factors and calculated E-values to examine the outcome of unmeasured confounders. We used cumulative mean values, based on repeated assessment of these factors, to reduce reporting bias.

This study also has several limitations. First, motor symptoms, such as tremor, rigidity, and dysphagia, could have implications for the ability or habit of food consumption and exercise; thus, an unhealthy diet and a sedentary lifestyle could be the outcome rather than the factors in disease progression and severity. To minimize this reverse causation, we restricted the main analyses to diet and physical activity before the diagnosis, when motor symptoms are less prominent and thus less likely to affect diet and physical activity, and we conducted additional analyses that left a lag time of 4 years between assessment of diet and physical activity and the PD diagnosis. Despite this precaution, a contribution of reverse causation cannot be totally excluded without a randomized clinical trial. Second, PD-specific deaths were not confirmed by neurologists, therefore the results should be viewed with caution. For other major causes of death, we identified 112 deaths from cardiovascular diseases and 69 deaths from cancers. Because of the limited number of cases of PD in this study, we were unlikely to achieve enough power to explore the association for deaths from other diseases except for PD. Third, participants in this study were predominantly of White race and had relatively higher socioeconomic status, which may limit the generalizability of the results to other populations.

## Conclusions

This cohort study showed that a better diet quality and a higher level of physical activity were inversely associated with mortality risk among men and women with PD. Consuming a high diet quality and engaging in physical activity or exercise could be targets for improved PD outcome. Reverse causation cannot be totally excluded, and the results need to be interpreted with caution.

## Notes

Supplement. **eTable 1.** Characteristics of Participants at the Last Diet Questionnaire Return Before Diagnosis of Parkinson Disease, According to Total Metabolic-Equivalent Hours per Week (MET-h/wk) **eTable 2.** Hazard Ratios (95% Confidence Intervals) for Diet Quality and Mortality Risk Among Individuals With Parkinson Disease, According to the Alternate Mediterranean Diet (aMED) **eTable 3.** Hazard Ratios (95% Confidence Intervals) for Diet Quality and Mortality Risk Among Individuals With Parkinson Disease, According to Cumulative Average Alternative Healthy Eating Index (AHEI) With Further Adjustment for Dietary Flavonoids Intake **eTable 4.** Hazard Ratios (95% Confidence Intervals) for Individual Dietary Component and Mortality Risk Among Individuals With Parkinson Disease **eTable 5.** Hazard Ratios (95% Confidence Intervals) for Diet Quality, Physical Activity, and Parkinson Disease-Specific Mortality Risk
