# Supplementary material for: Association of Diet and Physical Activity With All-Cause Mortality Among Adults With Parkinson Disease
Source: JAMA Netw Open. 2022 Aug 19;5(8):e2227738. doi: 10.1001/jamanetworkopen.2022.27738 (PMC9391952; doi:10.1001/jamanetworkopen.2022.27738)
Supplement: Supplement. — eTable 1. Characteristics of Participants at the Last Diet Questionnaire Return Before Diagnosis of Parkinson Disease, According to Total Metabolic-Equivalent Hours per Week (MET-h/wk) eTable 2. Hazard Ratios (95% Confidence Intervals) for Diet Quality and Mortality Risk Among Individuals With Parkinson Disease, According to the Alternate Mediterranean Diet (aMED) eTable 3. Hazard Ratios (95% Confidence Intervals) for Diet Quality and Mortality Risk Among Individuals With Parkinson Disease, According to Cumulative Average Alternative Healthy Eating Index (AHEI) With Further Adjustment for Dietary Flavonoids Intake eTable 4. Hazard Ratios (95% Confidence Intervals) for Individual Dietary Component and Mortality Risk Among Individuals With Parkinson Disease eTable 5. Hazard Ratios (95% Confidence Intervals) for Diet Quality, Physical Activity, and Parkinson Disease-Specific Mortality Risk [file jamanetwopen-e2227738-s001.pdf]

# Supplemental Online Content

Zhang X, Molsberry SA, Schwarzschild MA, Ascherio A, Gao X. Association of diet and physical activity with all-cause mortality among adults with Parkinson disease. *JAMA Netw Open*. 2022;5(8):e2227738. doi:10.1001/jamanetworkopen.2022.27738

**eTable 1.** Characteristics of Participants at the Last Diet Questionnaire Return Before Diagnosis of Parkinson Disease, According to Total Metabolic-Equivalent Hours per Week (MET-h/wk)

**eTable 2.** Hazard Ratios (95% Confidence Intervals) for Diet Quality and Mortality Risk Among Individuals With Parkinson Disease, According to the Alternate Mediterranean Diet (aMED)

**eTable 3.** Hazard Ratios (95% Confidence Intervals) for Diet Quality and Mortality Risk Among Individuals With Parkinson Disease, According to Cumulative Average Alternative Healthy Eating Index (AHEI) With Further Adjustment for Dietary Flavonoids Intake

**eTable 4.** Hazard Ratios (95% Confidence Intervals) for Individual Dietary Component and Mortality Risk Among Individuals With Parkinson Disease

**eTable 5.** Hazard Ratios (95% Confidence Intervals) for Diet Quality, Physical Activity, and Parkinson Disease-Specific Mortality Risk

This supplemental material has been provided by the authors to give readers additional information about their work.

© 2022 Zhang X et al. *JAMA Network Open.*

**eTable 1.** Characteristics of Participants at the Last Diet Questionnaire Return Before Diagnosis of Parkinson Disease, According to Total Metabolic-Equivalent Hours per Week (MET-h/wk)

| | **MET-h/wk** | | | |
|---|---|---|---|---|
| | **Quartile 1** | **Quartile 2** | **Quartile 3** | **Quartile 4** |
| **Health Professionals Follow-up Study** | | | | |
| N | 159 | 159 | 161 | 159 |
| Age at diagnosis, y* | 73.9 (9.2) | 72.4 (8) | 71.9 (8.7) | 72.6 (7.1) |
| Physical activity, MET-h/wk | 2.5 (1.9) | 11.9 (3.7) | 27.4 (5.7) | 70.6 (26.5) |
| Alternative Healthy Eating Index | 54.8 (12.6) | 57.4 (10.8) | 60.5 (11.8) | 61.8 (11.9) |
| Alternate Mediterranean diet | 3.8 (2) | 4.4 (1.6) | 4.8 (1.8) | 5.1 (1.6) |
| Smoking status | | | | |
| - Never, % | 85 (51.5) | 93 (57.7) | 94 (57.0) | 90 (55.0) |
| - Past, % | 69 (44.9) | 63 (41.1) | 65 (41.8) | 67 (43.5) |
| - Current, % | 5 (3.6) | 3 (1.2) | 2 (1.2) | 2 (1.6) |
| Body mass index, $kg/m^2$ | 26.4 (3.6) | 25.7 (2.7) | 25.3 (3) | 24.6 (2.4) |
| Total energy intake, kcal/d | 1943.2 (704.4) | 1889.5 (662.3) | 2032.4 (645.3) | 2094.6 (654.1) |
| Caffeine consumption, mg/d | 165.7 (212.3) | 117.8 (157.4) | 124.6 (148.6) | 137.9 (140.8) |
| Total flavonoids intake, mg/d | 297.5 (252.6) | 345.8 (230) | 344.8 (186.3) | 389.6 (272.3) |
| Use of NSAIDs[a], % | 46 (27.6) | 35 (21.8) | 47 (29.1) | 56 (33.8) |
| Hypertension, % | 71 (41.6) | 77 (48.4) | 66 (39.9) | 68 (43.8) |
| Diabetes mellitus, % | 13 (8.4) | 15 (11.9) | 15 (7.8) | 12 (7.1) |
| Hypercholesterolemia, % | 58 (34.6) | 70 (46.3) | 76 (46.5) | 75 (46.5) |
| **Nurses' Health Study** | | | | |
| N | 146 | 145 | 147 | 145 |
| Age at diagnosis, y* | 72.8 (8.1) | 71.4 (8.2) | 71.4 (7.3) | 71.9 (7) |
| Physical activity, MET-h/wk | 1.3 (0.9) | 6.3 (1.9) | 14.9 (3.7) | 39.7 (17) |
| Alternative Healthy Eating Index | 50.3 (10.8) | 54.3 (10.9) | 56.9 (11.2) | 58.7 (10.8) |
| Alternate Mediterranean diet | 3.5 (1.8) | 4.1 (2) | 4.5 (1.9) | 5.1 (1.7) |
| Smoking status | | | | |
| - Never, % | 80 (53.3) | 74 (48.7) | 84 (60.0) | 80 (57.4) |
| - Past, % | 55 (39.7) | 65 (47.5) | 60 (38.2) | 65 (42.6) |
| - Current, % | 11 (7.0) | 6 (3.8) | 3 (1.8) | 0 |
| Body mass index, $kg/m^2$ | 26.2 (4.7) | 26.1 (4.4) | 25.5 (4.3) | 25.3 (4.3) |
| Total energy intake, kcal/d | 1639.6 (530.7) | 1698.7 (546.5) | 1712.5 (529.1) | 1786.1 (503.9) |
| Caffeine intake, mg/d | 155.4 (169.9) | 164.6 (180.5) | 140.7 (188.4) | 149.3 (151.9) |
| Total flavonoids intake, mg/d | 310.5 (245) | 364.9 (320.5) | 342.6 (323.4) | 389.5 (323.1) |
| Use of NSAIDs[a], % | 38 (26.2) | 39 (26.7) | 47 (31.0) | 40 (25.2) |

© 2022 Zhang X et al. *JAMA Network Open.*

| | MET-h/wk | | | |
|---|---|---|---|---|
| | **Quartile 1** | **Quartile 2** | **Quartile 3** | **Quartile 4** |
| Hypertension, % | 76 (51.6) | 71 (49.8) | 74 (53.2) | 78 (56.1) |
| Diabetes mellitus, % | 17 (10.9) | 7 (5.2) | 8 (5.0) | 9 (5.5) |
| Hypercholesterolemia, % | 92 (61.1) | 90 (66.6) | 92 (64.7) | 85 (59.2) |
| Postmenopausal hormone use | | | | |
| - Premenopausal/never used, % | 58 (39.8) | 49 (32.1) | 43 (27.3) | 50 (38.3) |
| - Current user, % | 31 (20.5) | 44 (32.5) | 40 (24.2) | 33 (19.7) |
| - Past user, % | 57 (39.7) | 52 (35.3) | 64 (48.5) | 62 (42.0) |

Values are means (standard deviations) for continuous variables; numbers and percentages for categorical variables. Means, standard deviations, and percentages are standardized to the age distribution of the study population.

* Value is not age-standardized.

a Nonsteroidal anti-inflammatory drugs.

© 2022 Zhang X et al. *JAMA Network Open.*

**eTable 2.** Hazard Ratios (95% Confidence Intervals) for Diet Quality and Mortality Risk Among Individuals With Parkinson Disease, According to the Alternate Mediterranean Diet (aMED)

| | **aMED** | | | | |
|---|---|---|---|---|---|
| | **Quartile 1 (worst)** | **Quartile 2** | **Quartile 3** | **Quartile 4 (best)** | **p trend** |
| **Pre-diagnosis, cumulative average** | | | | | |
| **Health Professionals Follow-up Study** | | | | | |
| aMED, median (IQR) | 2.67 (2.00, 3.00) | 4.00 (3.80, 4.33) | 5.00 (5.00, 5.45) | 6.5 (6.00, 7.00) | |
| Case n | 132 | 132 | 135 | 130 | |
| Incidence, per 100,000 person-years | 3326 | 3367 | 3431 | 3291 | |
| Multivariable adjusted | 1 (ref) | 0.86 (0.65, 1.13) | 0.96 (0.73, 1.27) | 1.05 (0.77, 1.42) | 0.50 |
| **Nurses' Health Study** | | | | | |
| aMED, median (IQR) | 2.50 (2.00, 2.88) | 3.70 (3.40, 4.00) | 4.88 (4.50, 5.13) | 6.17 (5.82, 6.73) | |
| Case n | 94 | 106 | 110 | 103 | |
| Incidence, per 100,000 person-years | 2239 | 2533 | 2674 | 2445 | |
| Multivariable adjusted | 1 (ref) | 0.72 (0.53, 0.98) | 0.82 (0.60, 1.12) | 0.96 (0.69, 1.33) | 0.95 |
| **Pooled** | | | | | |
| Multivariable adjusted | 1 (ref) | 0.80 (0.65, 0.98) | 0.90 (0.73, 1.10) | 1.00 (0.80, 1.26) | 0.59 |
| **Post-diagnosis, cumulative average** | | | | | |
| **Health Professionals Follow-up Study** | | | | | |
| aMED, median (IQR) | 2.40 (2.00, 3.00) | 4.00 (3.75, 4.00) | 5.00 (4.67, 5.20) | 6.5 (6.00, 7.00) | |
| Case n | 153 | 112 | 109 | 89 | |
| Incidence, per 100,000 person-years | 9346 | 9939 | 7862 | 6279 | |
| Multivariable adjusted | 1 (ref) | 1.11 (0.82, 1.50) | 1.01 (0.74, 1.36) | 0.66 (0.48, 0.92) | 0.02 |
| **Nurses' Health Study** | | | | | |
| aMED, median (IQR) | 2.00 (1.17, 2.20) | 3.50 (3.00, 4.00) | 5.00 (4.50, 5.00) | 6.00 (6.00, 7.00) | |
| Case n | 111 | 114 | 73 | 70 | |
| Incidence, per 100,000 person-years | 8217 | 6445 | 5957 | 4789 | |
| Multivariable adjusted | 1 (ref) | 0.93 (0.68, 1.28) | 0.96 (0.64, 1.43) | 0.72 (0.46, 1.14) | 0.28 |
| **Pooled** | | | | | |
| Multivariable adjusted | 1 (ref) | 1.01 (0.81, 1.26) | 0.93 (0.72, 1.21) | 0.65 (0.48, 0.87) | 0.009 |

© 2022 Zhang X et al. *JAMA Network Open.*

Multivariable model was adjusted for age (year), smoking status (never smoked, <10 pack-years, 10-24 pack-years, 25-44 pack-years, or ≥45 pack-years), BMI (<21, 21-24.9, 25-29.9, or ≥30 $kg/m^2$), total energy intake (kcal/d, in quartiles), caffeine intake (mg/d, in quartiles), nonsteroidal anti-inflammatory drug use (yes or no), hypertension (yes or no), type 2 diabetes (yes or no), hypercholesterolemia (yes or no), and post-menopausal hormone use in women (pre-menopausal/never used, current user, or past user). Post-diagnosis models were further adjusted for pre-diagnosis aMED levels. IQR, interquartile range.

© 2022 Zhang X et al. *JAMA Network Open.*

**eTable 3.** Hazard Ratios (95% Confidence Intervals) for Diet Quality and Mortality Risk Among Individuals With Parkinson Disease, According to Cumulative Average Alternative Healthy Eating Index (AHEI) With Further Adjustment for Dietary Flavonoids Intake

| | **Alternative Healthy Eating Index** | | | | |
|---|---|---|---|---|---|
| | **Quartile 1** | **Quartile 2** | **Quartile 3** | **Quartile 4** | **p trend** |
| **Pre-diagnosis, cumulative average** | | | | | |
| HPFS | 1 (ref) | 0.82 (0.62, 1.09) | 0.75 (0.56, 1.00) | 0.85 (0.63, 1.16) | 0.36 |
| NHS | 1 | 0.75 (0.55, 1.02) | 0.85 (0.62, 1.15) | 0.61 (0.43, 0.84) | 0.009 |
| Pooled | 1 | 0.79 (0.64, 0.97) | 0.79 (0.64, 0.98) | 0.72 (0.52, 1.01) | 0.07 |
| **Post-diagnosis, cumulative average** | | | | | |
| HPFS | 1 (ref) | 0.88 (0.63, 1.22) | 0.88 (0.61, 1.28) | 0.58 (0.38, 0.89) | 0.006 |
| NHS | 1 | 0.96 (0.69, 1.34) | 0.81 (0.56, 1.19) | 0.62 (0.39, 0.99) | 0.03 |
| Pooled | 1 | 0.92 (0.73, 1.16) | 0.85 (0.65, 1.11) | 0.60 (0.44, 0.82) | <0.001 |

Model was adjusted for age (year), physical activity (MET-h/wk, in quartiles), smoking status (never smoked, <10 pack-years, 10-24 pack-years, 25-44 pack-years, or ≥45 pack-years), BMI (<21, 21-24.9, 25-29.9, or ≥30 kg/m$^2$), total energy intake (kcal/d, in quartiles), caffeine intake (mg/d, in quartiles), nonsteroidal anti-inflammatory drug use (yes or no), hypertension (yes or no), type 2 diabetes (yes or no), hypercholesterolemia (yes or no), and post-menopausal hormone use in women (pre-menopausal/never used, current user, or past user). Post-diagnosis models were further adjusted for pre-diagnosis levels.

© 2022 Zhang X et al. *JAMA Network Open.*

**eTable 4.** Hazard Ratios (95% Confidence Intervals) for Individual Dietary Component and Mortality Risk Among Individuals With Parkinson Disease

| | **Quartile 1** | **Quartile 2** | **Quartile 3** | **Quartile 4** | **p trend** |
|---|---|---|---|---|---|
| **Pre-diagnosis, cumulative average** | | | | | |
| **Vegetables** | | | | | |
| HPFS | 1 (ref) | 0.77 (0.58, 1.02) | 0.98 (0.73, 1.31) | 0.83 (0.60, 1.14) | 0.58 |
| NHS | 1 | 0.76 (0.56, 1.02) | 0.99 (0.73, 1.35) | 1.08 (0.77, 1.52) | 0.43 |
| Pooled | 1 | 0.76 (0.62, 0.94) | 0.98 (0.79, 1.21) | 0.94 (0.72, 1.22) | 0.95 |
| **Fruit** | | | | | |
| HPFS | 1 (ref) | 0.97 (0.72, 1.31) | 1.03 (0.76, 1.41) | 1.16 (0.83, 1.64) | 0.26 |
| NHS | 1 | 0.88 (0.64, 1.21) | 0.78 (0.55, 1.10) | 0.91 (0.63, 1.31) | 0.50 |
| Pooled | 1 | 0.93 (0.74, 1.15) | 0.91 (0.69, 1.19) | 1.04 (0.81, 1.33) | 0.76 |
| **Whole grains** | | | | | |
| HPFS | 1 (ref) | 0.81 (0.60, 1.07) | 0.79 (0.59, 1.04) | 0.62 (0.47, 0.83) | 0.003 |
| NHS | 1 | 0.59 (0.42, 0.82) | 0.47 (0.34, 0.65) | 0.49 (0.35, 0.68) | <0.001 |
| Pooled | 1 | 0.70 (0.51, 0.94) | 0.61 (0.37, 1.02) | 0.56 (0.45, 0.71) | <0.001 |
| **Sugar-sweetened beverages and fruit juice** | | | | | |
| HPFS | 1 (ref) | 0.69 (0.50, 0.95) | 0.78 (0.61, 1.00) | 0.85 (0.66, 1.11) | 0.66 |
| NHS | 1 | 0.75 (0.55, 1.03) | 0.77 (0.57, 1.05) | 0.74 (0.53, 1.04) | 0.40 |
| Pooled | 1 | 0.72 (0.58, 0.90) | 0.77 (0.64, 0.94) | 0.81 (0.66, 1.00) | 0.37 |
| **Nut and legumes** | | | | | |
| HPFS | 1 (ref) | 0.79 (0.59, 1.04) | 0.76 (0.57, 1.01) | 0.77 (0.56, 1.05) | 0.16 |
| NHS | 1 | 1.03 (0.76, 1.39) | 0.97 (0.72, 1.30) | 0.79 (0.57, 1.09) | 0.11 |
| Pooled | 1 | 0.89 (0.69, 1.17) | 0.85 (0.68, 1.08) | 0.78 (0.62, 0.98) | 0.04 |
| **Red/processed meat** | | | | | |
| HPFS | 1 (ref) | 1.06 (0.80, 1.41) | 0.97 (0.71, 1.33) | 0.80 (0.58, 1.10) | 0.09 |
| NHS | 1 | 0.65 (0.47, 0.90) | 0.60 (0.43, 0.85) | 0.59 (0.42, 0.84) | 0.01 |
| Pooled | 1 | 0.84 (0.52, 1.35) | 0.77 (0.48, 1.23) | 0.69 (0.52, 0.92) | 0.004 |
| **Trans fat** | | | | | |
| HPFS | 1 (ref) | 0.73 (0.55, 0.96) | 0.85 (0.65, 1.12) | 0.92 (0.69, 1.24) | 0.68 |
| NHS | 1 | 0.68 (0.49, 0.95) | 0.61 (0.44, 0.85) | 0.63 (0.46, 0.87) | 0.006 |
| Pooled | 1 | 0.71 (0.57, 0.88) | 0.73 (0.53, 1.01) | 0.77 (0.53, 1.11) | 0.21 |
| **Long-chain n-3 fatty acids** | | | | | |
| HPFS | 1 (ref) | 1.21 (0.92, 1.59) | 1.21 (0.85, 1.72) | 1.61 (1.23, 2.11) | 0.001 |
| NHS | 1 | 1.08 (0.81, 1.44) | 0.90 (0.67, 1.21) | 1.00 (0.73, 1.39) | 0.78 |
| Pooled | 1 | 1.15 (0.94, 1.40) | 1.03 (0.77, 1.37) | 1.28 (0.81, 2.04) | 0.40 |
| **PUFA** | | | | | |
| HPFS | 1 (ref) | 0.84 (0.64, 1.10) | 0.74 (0.57, 0.97) | 1.00 (0.75, 1.32) | 0.93 |

© 2022 Zhang X et al. *JAMA Network Open.*

| | | | | | |
|---|---|---|---|---|---|
| NHS | 1 | 0.76 (0.57, 1.01) | 1.02 (0.77, 1.35) | 0.91 (0.66, 1.25) | 0.92 |
| Pooled | 1 | 0.80 (0.66, 0.97) | 0.87 (0.64, 1.18) | 0.95 (0.77, 1.18) | 0.99 |
| **Sodium** | | | | | |
| HPFS | 1 (ref) | 0.99 (0.71, 1.38) | 1.00 (0.67, 1.52) | 0.87 (0.54, 1.40) | 0.66 |
| NHS | 1 | 0.83 (0.56, 1.23) | 0.99 (0.65, 1.52) | 0.85 (0.51, 1.39) | 0.69 |
| Pooled | 1 | 0.92 (0.71, 1.18) | 1.00 (0.74, 1.35) | 0.86 (0.61, 1.21) | 0.56 |
| **Alcohol** | | | | | |
| HPFS | 1 (ref) | 0.83 (0.62, 1.09) | 0.64 (0.49, 0.84) | 0.82 (0.61, 1.10) | 0.05 |
| NHS | 1 | 0.86 (0.65, 1.15) | 0.92 (0.68, 1.24) | 1.09 (0.80, 1.48) | 0.35 |
| Pooled | 1 | 0.84 (0.69, 1.03) | 0.77 (0.54, 1.09) | 0.94 (0.71, 1.24) | 0.76 |

Multivariable model was adjusted for age (year), smoking status (never smoked, <10 pack-years, 10-24 pack-years, 25-44 pack-years, or ≥45 pack-years), BMI (<21, 21-24.9, 25-29.9, or ≥30 kg/$m^2$), total energy intake (kcal/d, in quartiles), caffeine intake (mg/d, in quartiles), nonsteroidal anti-inflammatory drug use (yes or no), hypertension (yes or no), type 2 diabetes (yes or no), hypercholesterolemia (yes or no), and post-menopausal hormone use in women (pre-menopausal/never used, current user, or past user).

© 2022 Zhang X et al. *JAMA Network Open.*

**eTable 5.** Hazard Ratios (95% Confidence Intervals) for Diet Quality, Physical Activity, and Parkinson Disease-Specific Mortality Risk

| | **Quartile 1** | **Quartile 2** | **Quartile 3** | **Quartile 4** | **p trend** |
|---|---|---|---|---|---|
| **Alternative Healthy Eating Index** | | | | | |
| **Pre-diagnosis, cumulative average** | | | | | |
| HPFS | 1 (ref) | 0.80 (0.55, 1.16) | 0.81 (0.56, 1.17) | 0.74 (0.50, 1.11) | 0.22 |
| NHS | 1 | 0.81 (0.53, 1.25) | 0.85 (0.54, 1.31) | 0.76 (0.49, 1.19) | 0.27 |
| Pooled | 1 | 0.80 (0.61, 1.07) | 0.82 (0.62, 1.09) | 0.75 (0.56, 1.01) | 0.10 |
| **Post-diagnosis, cumulative average** | | | | | |
| HPFS | 1 (ref) | 0.80 (0.52, 1.23) | 0.71 (0.43, 1.17) | 0.42 (0.24, 0.75) | 0.002 |
| NHS | 1 | 0.92 (0.58, 1.47) | 0.84 (0.49, 1.45) | 0.67 (0.35, 1.27) | 0.14 |
| Pooled | 1 | 0.85 (0.62, 1.17) | 0.77 (0.53, 1.11) | 0.52 (0.33, 0.80) | <0.001 |
| **Metabolic-equivalent hour per week** | | | | | |
| **Pre-diagnosis, cumulative average** | | | | | |
| HPFS | 1 (ref) | 1.06 (0.73, 1.55) | 1.29 (0.89, 1.87) | 0.87 (0.59, 1.29) | 0.47 |
| NHS | 1 | 0.74 (0.48, 1.14) | 0.72 (0.47, 1.10) | 0.92 (0.60, 1.41) | 0.80 |
| Pooled | 1 | 0.90 (0.63, 1.28) | 0.97 (0.55, 1.72) | 0.89 (0.67, 1.19) | 0.59 |
| **Post-diagnosis, cumulative average** | | | | | |
| HPFS | 1 (ref) | 1.11 (0.75, 1.64) | 0.70 (0.45, 1.08) | 0.38 (0.22, 0.64) | <0.001 |
| NHS | 1 | 0.52 (0.33, 0.81) | 0.51 (0.31, 0.83) | 0.36 (0.20, 0.66) | 0.007 |
| Pooled | 1 | 0.76 (0.36, 1.61) | 0.60 (0.43, 0.84) | 0.37 (0.25, 0.55) | <0.001 |

Multivariable model was adjusted for age (year), smoking status (never smoked, <10 pack-years, 10-24 pack-years, 25-44 pack-years, or ≥45 pack-years), BMI (<21, 21-24.9, 25-29.9, or ≥30 $kg/m^2$), total energy intake (kcal/d, in quartiles), caffeine intake (mg/d, in quartiles), nonsteroidal anti-inflammatory drug use (yes or no), hypertension (yes or no), type 2 diabetes (yes or no), hypercholesterolemia (yes or no), and post-menopausal hormone use in women (pre-menopausal/never used, current user, or past user). Post-diagnosis models were further adjusted for pre-diagnosis levels.

© 2022 Zhang X et al. *JAMA Network Open.*
